# Jungle Giants: Assessing Sustainable Harvesting in a Difficult-to-Survey Species (*Python reticulatus*)

**DOI:** 10.1371/journal.pone.0158397

**Published:** 2016-07-08

**Authors:** Daniel J. D. Natusch, Jessica A. Lyons, Mumpuni, Awal Riyanto, Richard Shine

**Affiliations:** 1 School of Life and Environmental Sciences, University of Sydney, Sydney, New South Wales, Australia; 2 Resource Evaluation and Development, Bamaga, Queensland, Australia; 3 Museum Zoologicum Bogoriense Research Centre for Biology, Indonesian Institute of Sciences, Cibinong, Bogor, Indonesia; The University of Texas Arlington, UNITED STATES

## Abstract

Sustainability of wildlife harvests is critical but difficult to assess. Evaluations of sustainability typically combine modelling with the measurement of underlying abundances. For many taxa harvested in developing countries, however, abundances are near-impossible to survey and a lack of detailed ecological information impedes the reliability of models. In such cases, repeated surveys of the attributes of harvested individuals may provide more robust information on sustainability. If the numbers, sizes and other demographic attributes of animals taken for the commercial trade do not change over biologically significant time intervals (decades), there is a *prima facie* case that the harvest is indeed sustainable. Here, we report the results of examinations of > 4,200 reticulated pythons (*Python reticulatus*) taken for the commercial leather industry in northern and southern Sumatra, Indonesia. The numbers, mean body sizes, clutch sizes, sizes at maturity and proportion of giant specimens have not decreased between our first surveys (1995) and repeat surveys (2015). Thus, despite assumptions to the contrary, the harvest appears to be sustainable. We use our data to inform the design of future monitoring programs for this species. Our study underpins the need for robust science to inform wildlife trade policy and decision-making, and urges wildlife managers to assess sustainability of difficult-to-survey terrestrial wildlife by drawing inferences directly from the harvest itself.

## Introduction

Most people agree that levels of commercial exploitation of natural resources should be controlled so as to be ecologically sustainable over long time periods [1]. Enormous effort has been dedicated to achieving this goal for a range of taxa, and sustainability has become a cornerstone criterion for modern conservation biology [2,3]. But this goal has been difficult to achieve, commonly because of difficulties in striking a balance between the intrinsic biological value of a resource, and the economic benefits that can be derived from it [4,5].

One fundamental problem is the challenge of identifying what level of harvest is indeed sustainable. In particular, many wildlife taxa that are intensively harvested are poorly known ecologically, because they occur in parts of world where infrastructural support is meagre, and their natural history makes field surveys virtually impossible [6]. For example, it can be remarkably difficult to accurately census the populations of cryptic species, or those living in habitats where logistical constraints are high and detectability is poor (e.g., swamps or rainforests) [7]. Nonetheless, given the increase in global wildlife trade and corresponding species declines, we urgently require techniques by which sustainable levels of harvesting can be determined for even the most challenging conditions.

Typically, sustainable offtake is estimated by quantifying the number of individuals in a population; if we know what proportion of the resource is being removed, we can predict the impact of various harvest intensities on population density [6]. But establishing baseline densities is near-impossible for some kinds of organisms, creating considerable uncertainty in sustainability assessments based on this method. As an alternative, wildlife managers can assess the influence of harvesting on the broader population by drawing inferences from the harvest itself. For example, a harvest that focuses primarily on immature males is more likely to be sustainable than one that focuses on reproductive females.

Such a “snapshot” assessment, based upon the attributes of harvested animals at one point in time, necessarily is prone to error as a basis for predicting longer-term sustainability. A far more robust approach is to repeat surveys of harvested animals through time, to identify temporal shifts (if any) in the size and nature of the offtake [8,9]. For example, fisheries managers use sophisticated mathematical models (often based on catch per unit effort) to assess the level of offtake that is sustainable. The most obvious indicator of unsustainable offtake is a decline in absolute numbers of individuals harvested through time (despite constant catching effort), but demographic changes such as skewed sex ratios, reduction in mean body sizes, sizes at maturity or fecundity are also commonly used to reveal harvest impacts on populations [10,11]. Unfortunately, these techniques are rarely applied to the management and determination of sustainability for terrestrial wildlife harvests, and if they are, they are rarely extended to tropical developing countries [9,12].

We used repeated surveys of harvested animals, conducted 20 years apart, to evaluate the sustainability of trade in the world’s longest species of snake, the reticulated python (*Python reticulatus*). The commercial trade in reticulated pythons began in the mid-1930s, and by the 1960s hundreds of thousands of individual pythons were being harvested every year for their meat and skins [13,14]. Today, more than 300,000 wild reticulated pythons are harvested from Indonesia and Malaysia annually [15,16]. International trade in reticulated pythons is regulated by the Convention on International Trade in Endangered Species of Wild Fauna and Flora (CITES). Parties to CITES are required under Article IV to complete a non-detriment finding before exports of python skins can commence, to ensure that trade does not imperil the survival of the species in the wild [17]. Despite these provisions, the sustainability of trade in reticulated pythons has been challenged based on the large volume of trade, coupled with anecdotal information on the ecological characteristics of the species [16]. Those concerns have resulted in trade restrictions, such as declining harvest quotas and trade bans, which have negatively affected the livelihood benefits that local people derive from trade [16,18]. In reality, little objective information exists about whether or not their trade is sustainable.

**Fig 1:**
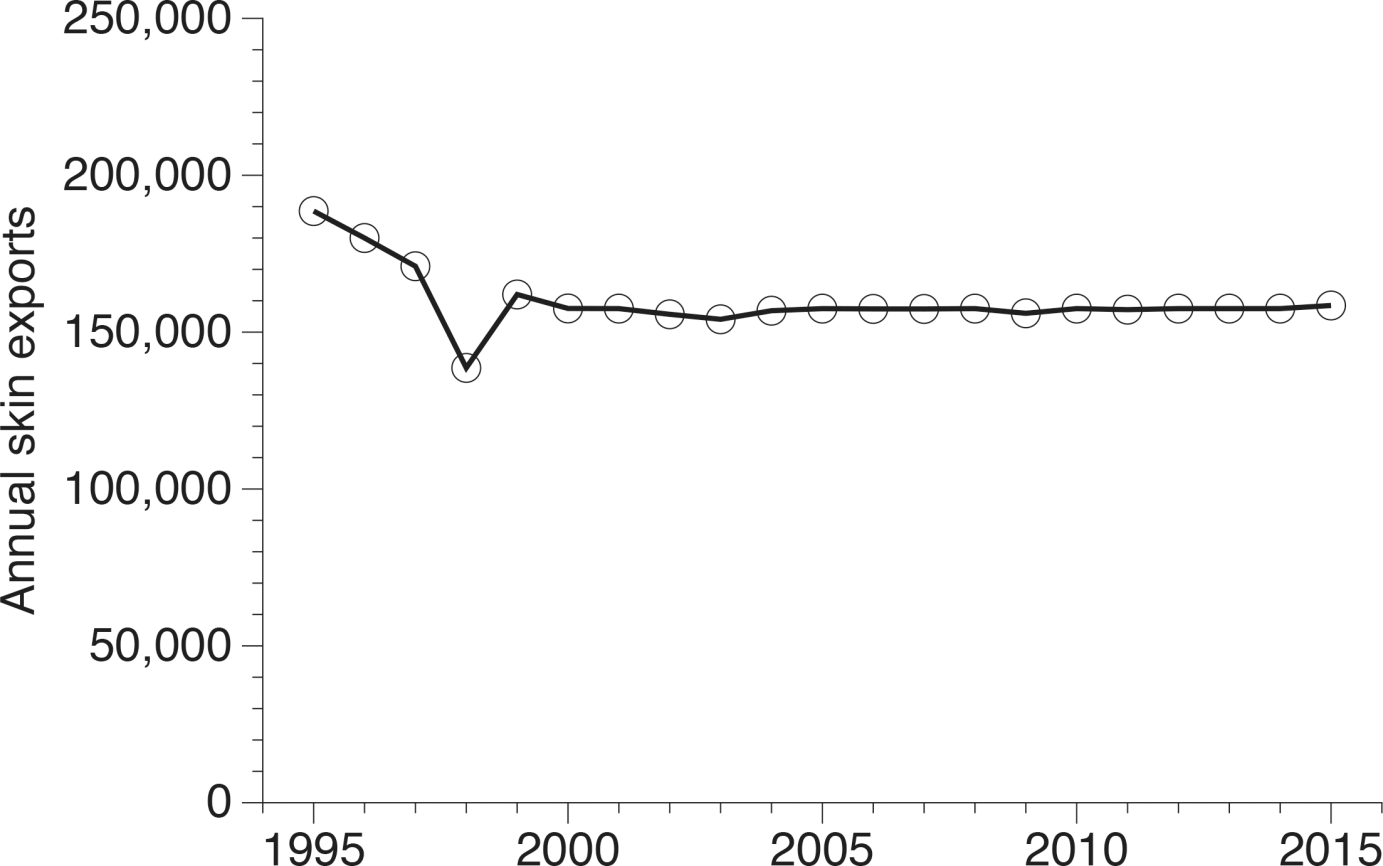
Annual exports of reticulated python skins from Indonesia between 1995 and 2015. From 1995 to 2000 the annual export quota ranged between 138,600 and 189,000 skins, but was lowered to 157,500 since 2001. Note that annual export quotas are 10% lower than annual harvest quotas, with the remaining 10% being allocated to domestic trade. Source: Indonesian CITES Management Authority and UNEP-WCMC CITES Trade Database.

Obtaining the types of information required to make robust assessments of the status of reticulated python populations is remarkably difficult. These animals are highly cryptic, remaining concealed for long periods in inaccessible habitats, which makes traditional field-based estimates of population sizes near-impossible [6,7]. As an alternative, between 1993 and 1996 we visited python processing facilities in northern and southern Sumatra, Indonesia, and gathered data on the biology of harvested snakes [7, 19,20]. In 2014 and 2015 we returned to those same facilities to collect the same types of data and draw inferences about the impact of harvest on the reticulated python population over the intervening 20-year period. Northern and southern Sumatra have traditionally been major harvest areas for this species [13]. Harvesting is regulated using a quota system, which limits the total number of individuals that can be captured and exported each year (to approximately 175,500 skins; Fig 1). The national quota is then divided among the provinces based on application by companies wishing to trade in pythons. Quotas for the capture of pythons in northern and southern Sumatra have been issued every year between 1995 and 2015, and national exports have remained constant, allowing us to examine the impact of high offtake over the last 20 years [13,14,15,21,22; Fig 1]. We hypothesised that if the harvest was unsustainable over that period we would expect our data to reveal a > 15% decline in (1) the number of snakes brought to processing facilities, (2) mean body sizes and proportion of giant specimens, (3) sizes at maturation, and (4) fecundity. The present paper compares the recently studied pythons with those examined 20 years ago, and uses the combined dataset to assess the ecological sustainability of the commercial trade in this species and inform future monitoring strategies. We conclude by urging wildlife managers working on difficult-to-survey terrestrial species (e.g., snakes) to focus sustainability assessments on inferences drawn from the harvest itself.

## Materials and Methods

### Ethics statement

Our data were gathered from snakes harvested for a commercial industry, which employs humane methods of killing for reptiles in the skin trade (brain destruction; see [23]). We stress that no snakes were harmed for the purpose of our study. We merely utilised an existing trade. All work was carried out with relevant permissions and permits (278/SIP/FRP/SM/IX/2014 and 311/SIP/FRP/SM/X/2014) from the Ministry of Research and Technology of the Republic of Indonesia.

### Study sites and general protocols

In both northern and southern Sumatra, Indonesia, reticulated pythons are captured in the wild and brought to local processing facilities to be killed and skinned. We visited processing facilities in both of these provinces between 1993 and 1996 and again in 2014 and 2015 (hereafter referred to as the 1995 and 2015 survey periods). The ways in which pythons were captured and the characteristics of the study sites are described elsewhere [7,19]. During both survey periods we visited each processing facility four times, with our trips spread evenly throughout the year to encompass seasonal variation in our samples of harvested pythons. The processing facilities visited during the two survey periods were identical (the facilities are still operated in the same ways by the same owners), but in 2015 we visited additional facilities (snakes from those premises are included in our analysis of harvest demographics but not trade volumes). At each facility we examined freshly-killed pythons before and after skinning. We recorded snout-vent length (SVL) and body mass, and then examined the snake’s carcasses to determine sex and reproductive condition (by visual inspection of gonads). Finally, we determined clutch sizes by counting eggs within the oviducts of female pythons.

### Data comparisons

We compared the number of pythons processed per day at facilities in 1995 with the same facilities in 2015. Although we visited additional facilities in 2015, these were not included in our comparison (to avoid biases due to differences among facilities). Because we often visited more than one facility in a day, we averaged the number of pythons processed at each facility to determine the number of pythons per facility day.

Our comparison of sizes at sexual maturity focused on female snakes, as they are more critical for population persistence, and because 99% of males in our second sample were mature. We classified females as mature if they had thickened muscular oviducts, vitellogenic ovarian follicles (depending on size and colouration), and primary follicles larger than 8 mm in diameter and/or corpora albicantia from previous reproductive events. To assess changes in the size at sexual maturity of female pythons we determined the SVL at which 50% of females reach maturity (hereafter SVL_50_) for each study period and location. We did this by grouping the proportions of mature pythons into 10 cm length cohorts, which was best described (evaluated using Akaike Information Criterion) by a two-parameter logistic function:

$$P_{M}={\left[1+e\left(-a\left(L-b\right)\right)\right]}^{-1}$$

where *P*_*M*_ = estimated proportion of mature pythons, *L* = SVL of pythons (cm) and *a* and *b* = coefficients that define the shape and position of the fitted curve. We used JMP Pro 11 (SAS Institute Cary NC) to calculate the observed mature proportion, its predicted probability and coefficients of the logistic equation. We used JMPs negative ratio tool to estimate SVL_50_ by substituting *P*_*M*_ = 0.5 into the equation above and solving for *L*. Data were log-transformed where necessary to meet assumptions of normality and homogeneity of variance.

### Sustainability criteria and statistical power

We considered an effect size of 0.15 (a 15% decline—or 0.75% annually—in the attributes examined) as the criterion for determining whether the harvest is unsustainable or not. Because accurate knowledge of population change is vital for management and decision-making, we performed a post-hoc statistical power analysis to calculate the probability of making a type II error when interpreting our results (falsely detecting no change in the attributes of pythons when such a change has actually occurred) [24,25]. In addition, we simulated the number of snakes required to be examined during future monitoring events to detect declines (of various magnitudes) with sufficient statistical power. We performed all analyses in G*Power [26] and considered 0.8 to be sufficient statistical power in all tests [27].

## Results

### Number of snakes

The number of snakes processed per day varied among study sites on both survey occasions, with more snakes processed in northern than southern Sumatra (*F*_2,149_ = 14.8, *P* <0.0001, Table 1). The number of snakes processed per day between survey occasions was not significantly different, but averaged higher in 2015 than 1995 (*F*_2,149_ = 3.84, *P* = 0.052). A chi-squared test on pooled data showed that sex ratios were similar between sites and survey periods (mean 52% males in 1995 vs. 53% males in 2015; χ^2^ = 1.07, df = 3, *P* = 0.78).

**Table 1:** Numbers and body sizes of reticulated pythons examined at the same facilities in Sumatra, Indonesia, in 1995 and 2015. Body sizes are calculated for the total sample from all facilities visited in both survey periods, whereas numbers of snakes represent only those recorded from the facilities that were visited during both the survey periods.

Site	Year	# facility days	# pythons recorded	Mean pythons/day (se)	Mean SVL (se)	Mean mass (se)	% > 450 cm SVL
North	1995	22	664	29.7 (2.1)	252.4 (2.1)	6835 (228)	1.40%
North	2015	30	1001	33.4 (1.8)	281.8 (1.8)	7822 (184)	1.07%
South	1995	44	1006	22.9 (1.5)	245.5 (1.8)	5762 (218)	1.37%
South	2015	55	1360	25.2 (1.3)	305.9 (1.6)	10109 (157)	2.05%

### Body sizes

**Fig 2:**
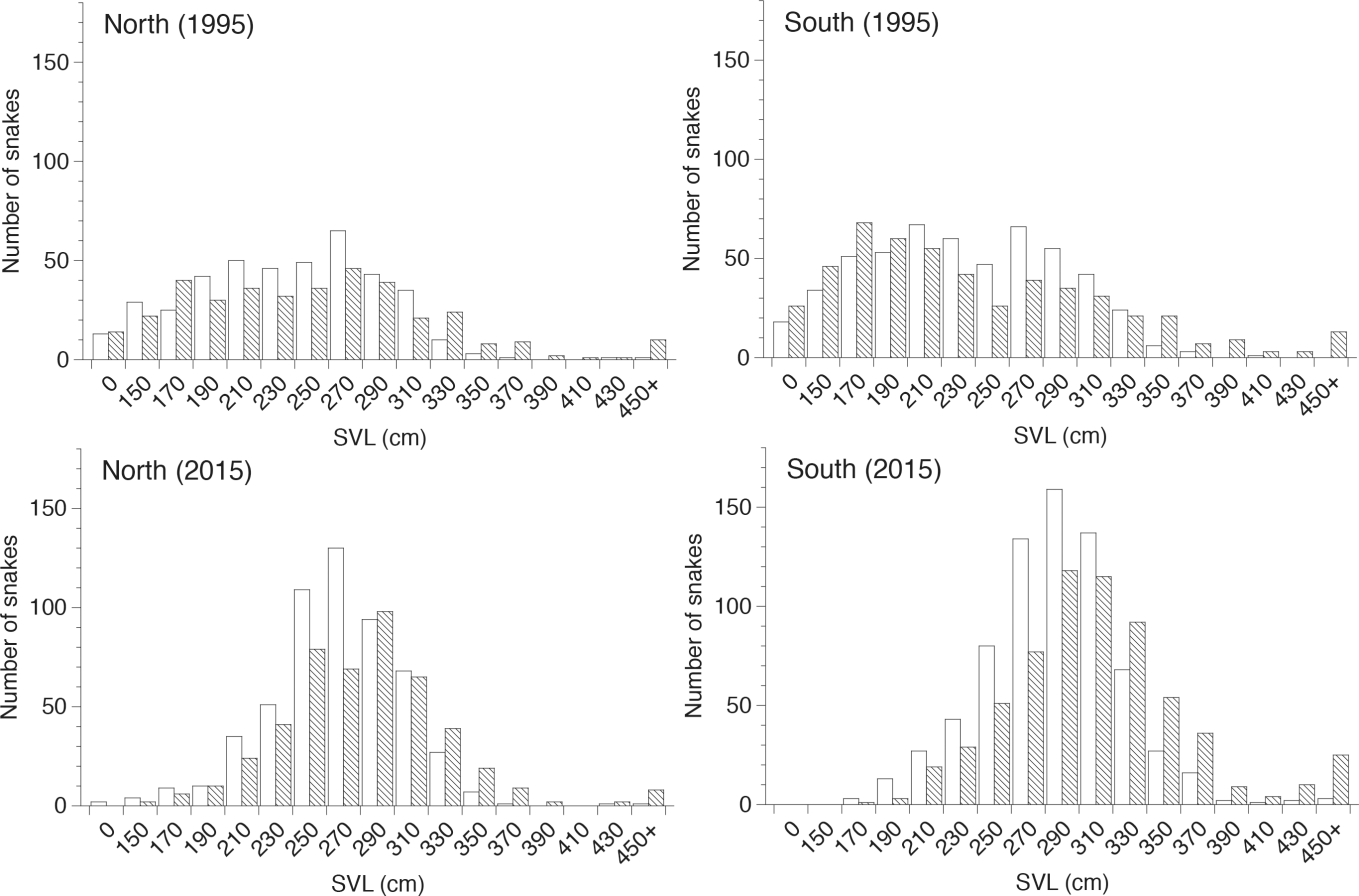
Comparison of body size distributions of male (hollow columns) and female (hatched columns) reticulated pythons harvested for trade in northern and southern Sumatra, Indonesia, in 1995 and 2015.

The pythons harvested for trade averaged larger in 2015 than in 1995 (Fig 2). A two-way analysis of variance (ANOVA) with study period and site as factors and *ln* SVL as the dependent variable confirmed this pattern, revealing a significant interaction whereby SVL of pythons was greater in 2015 than 1995 at both sites, and in the 2015 study snakes from southern Sumatra were longer than those in the north (interaction term *F*_3,4214_ = 72.7, *P* = <0.0001, Table 1). We repeated the analysis separately for males and females, with the same result (males, *F*_3,2213_ = 27.7, *P* < 0.0001; females, *F*_3,1991_ = 46.5, *P* < 0.0001). In addition to changes in mean body sizes due to harvesting, we should also expect to see the proportion of truly giant snakes in our samples (those larger than 450 cm SVL) decrease over time. Our results do not support this conclusion, and show that giant snakes are equally common between survey periods (χ^2^ = 5.31, df = 3, *P* = 0.15, Table 1).

To compare the body mass of harvested pythons we repeated the ANOVA above but with *ln* mass as the dependent variable. A significant interaction revealed that pythons were heavier in 2015 than 1995, but the site with the heaviest snakes changed between survey periods (from northern Sumatra in 1995 to southern Sumatra in 2015; *F*_3,3658_ = 112, *P* <0.0001). But is this difference because pythons have become heavier over the 20-year period at certain sites, or is it simply related to the overall difference in SVL of pythons between surveys? To answer this we included *ln* SVL as a covariate in a two-way analysis of covariance (ANCOVA) with site and survey as factors and *ln* body mass as the dependant variable. A significant interaction between site and survey showed that pythons from northern Sumatra were heavier relative to their SVL than at other sites, but only when surveyed in 1995 (*F*_4,3654_ = 15.2, *P* < 0.0001).

### Size at maturity

**Fig 3:**
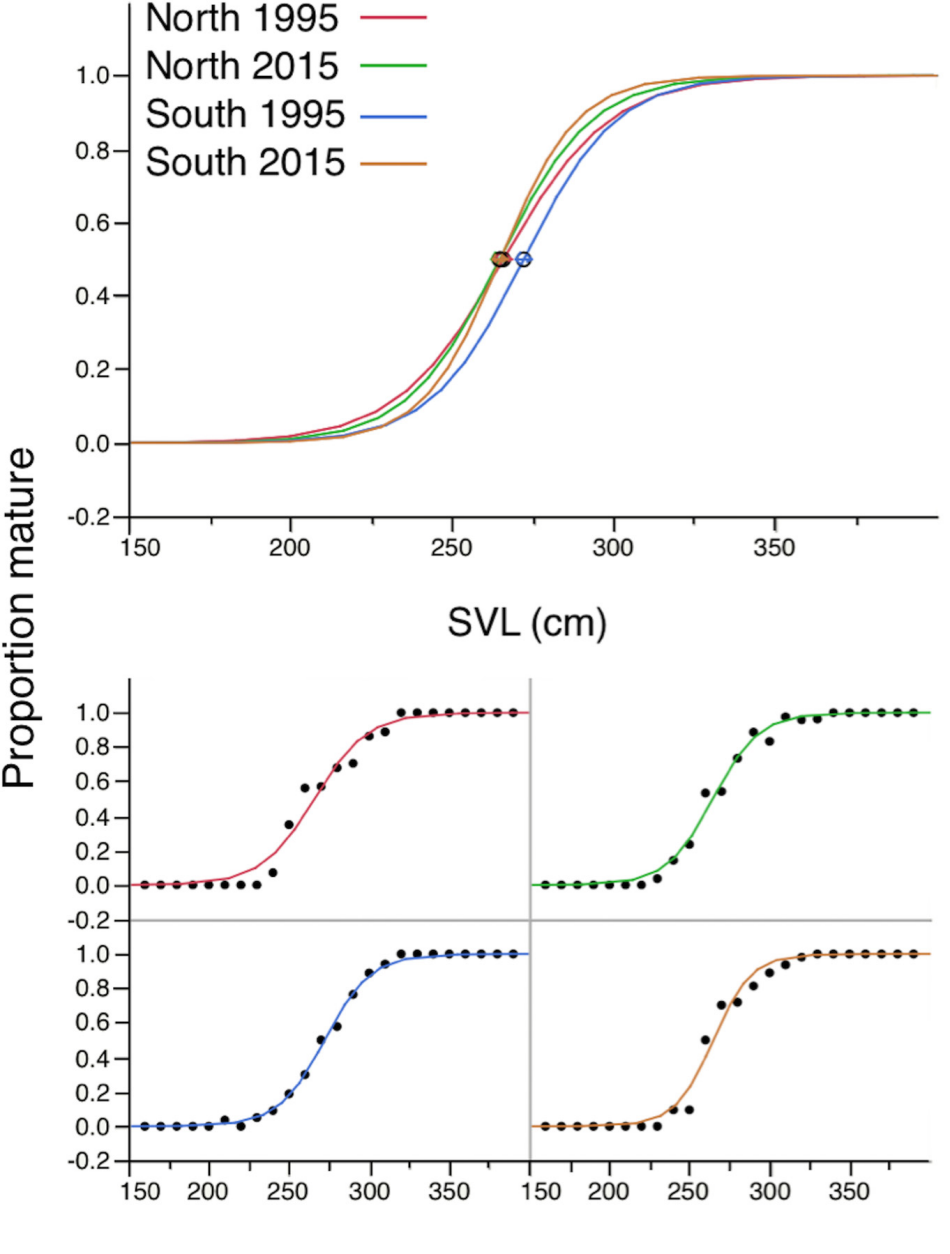
Proportion of mature (*P*_*M*_) reticulated pythons by 10 cm length classes harvested for trade in northern and southern Sumatra, Indonesia, in 1995 and 2015.

The proportion of reproductive females at different sizes was broadly similar between the two survey periods at both sites (Fig 3). SVL_50_ showed little change except for snakes in southern Sumatra, where it had decreased by approximately 8 cm between 1995 and 2015 (Table 2). The size of the smallest mature snakes showed remarkably little variation among sites or between survey periods (Table 2).

**Table 2:** Predicted snout-vent lengths (SVL) at which 50% of female reticulated python are sexually reproductive at two sites in Sumatra, Indonesia, based on surveys conducted 20 years apart.

Year	Site	Smallest mature female	Predicted SVL_50_	Std error	Lower 95% CI	Upper 95% CI
1995	North	244	265.8	1.36	263.2	268.5
2015	North	239	264.7	1.26	262.2	267.2
1995	South	230	272.2	1.26	269.7	274.7
2015	South	224	264.9	1.15	262.7	267.2

### Fecundity

**Fig 4:**
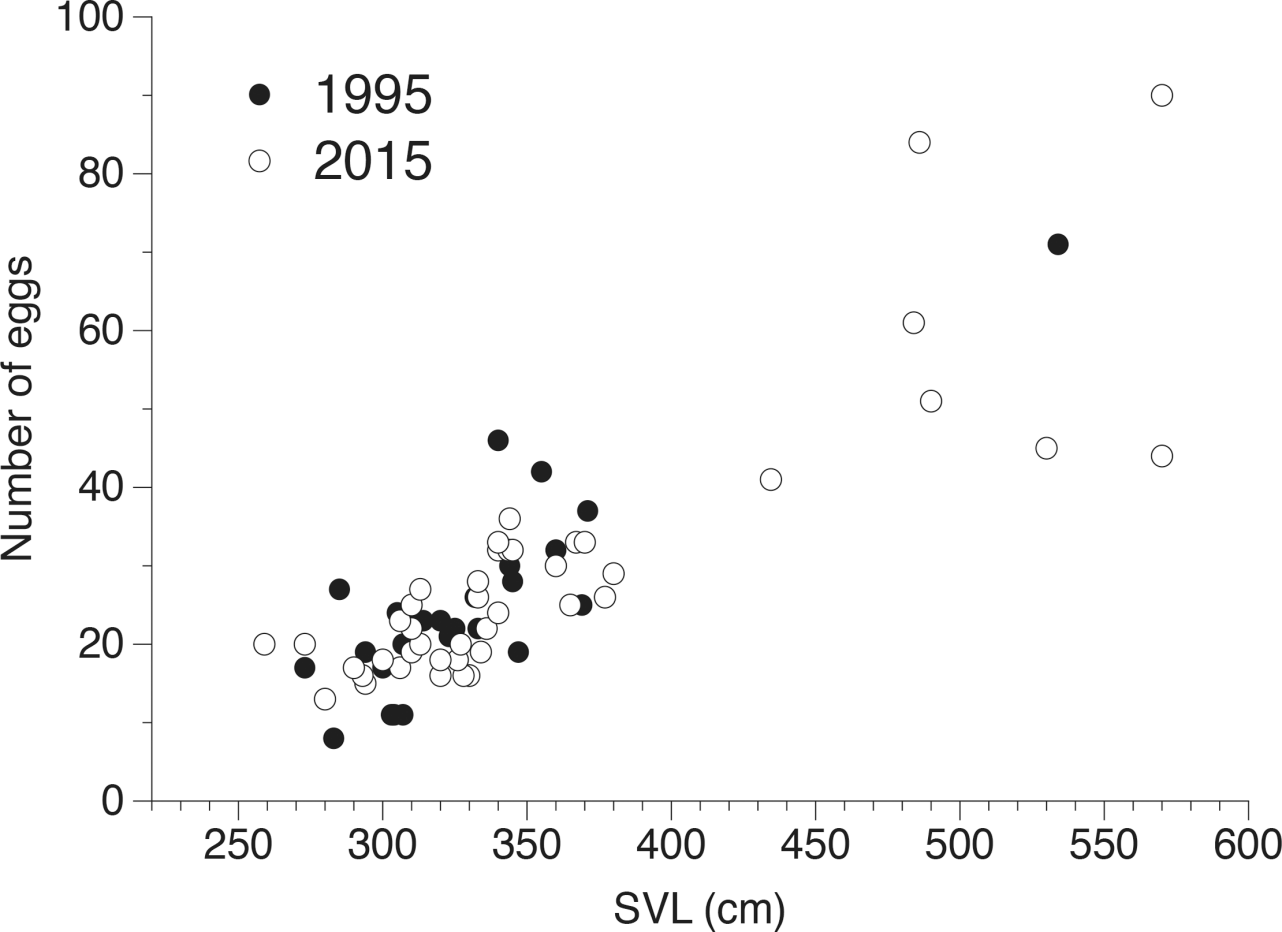
Relationship between body sizes and clutch sizes of female reticulated pythons from Sumatra, Indonesia, in 1995 and 2015. Data from northern and southern Sumatra have been pooled.

After deletion of a non-significant two-way interaction term, ANCOVA (with survey period and site as factors, *ln* clutch size as the dependant variable and *ln* SVL as the covariate) showed that clutch sizes of snakes relative to their SVL had not changed between survey periods (*F*_3, 69_ = 0.02, *P* = 0.88; Fig 3), and although averaging higher in southern than northern Sumatra, the difference was not great enough to be significant (*F*_3,69_ = 3.19, *P* = 0.079; Fig 4).

### Statistical power

**Fig 5:**
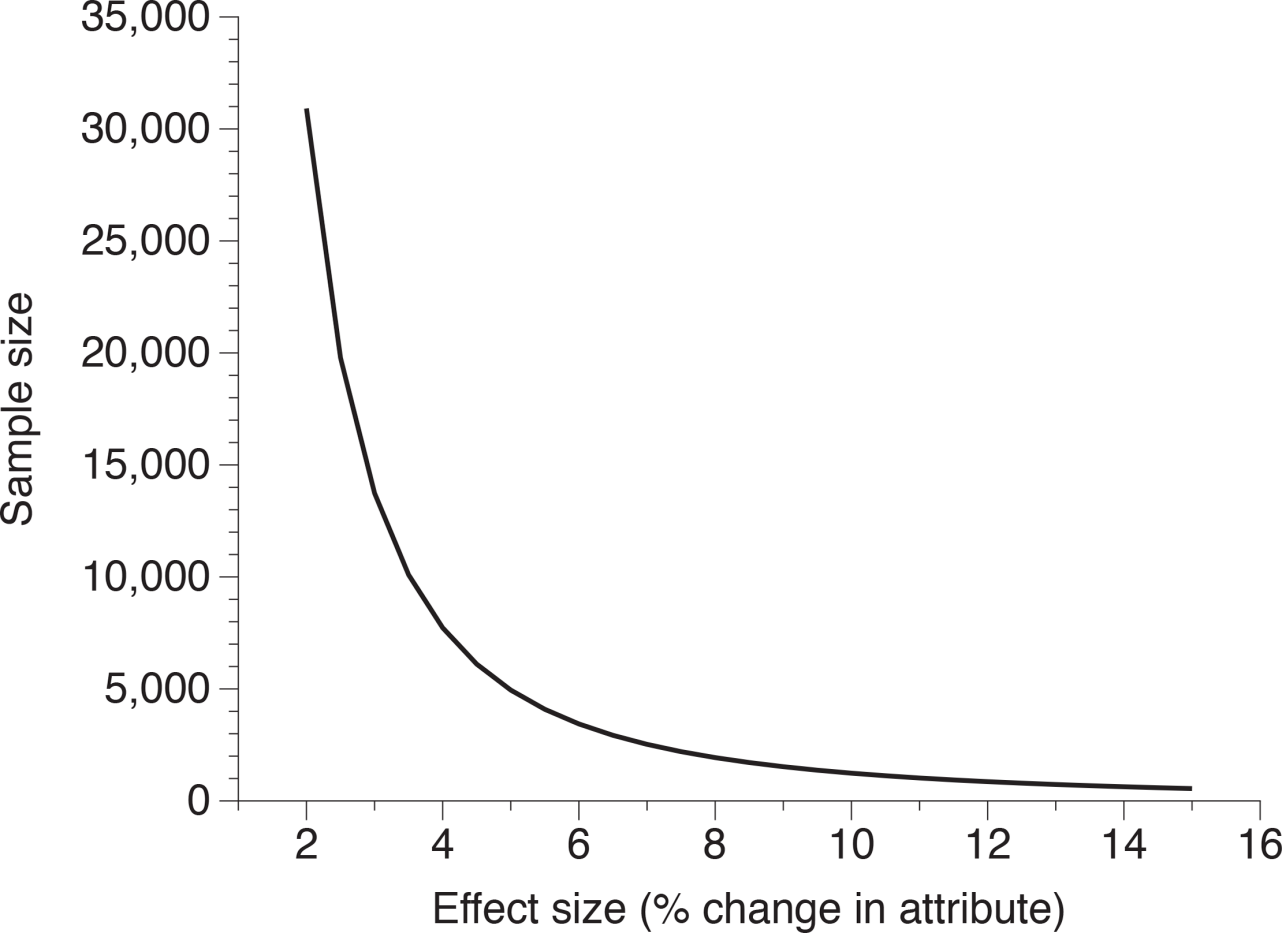
Minimum sample sizes of reticulated pythons (*Python reticulatus*) needed to detect effect sizes (changes) of various magnitudes with sufficient statistical power (> 0.8). Smaller effect sizes require much larger sample sizes.

Our post-hoc test showed that we achieved sufficient statistical power to support the conclusion that any change in the attributes of pythons we measured was less than our *a priori* criterion of 15% (powers of 0.98 and 0.94 for southern and northern Sumatra, respectively). However, the sample sizes required to detect changes with sufficient power rapidly increases with decreasing effect size (Fig 5). For example, our statistical power to detect a 10% (rather than 15%) change would be lower (power = 0.78).

## Discussion

Despite two decades of harvesting since our first survey, attributes of the commercial harvest of reticulated pythons were strikingly similar. Over the past 20 years, nearly one million reticulated pythons have been removed from the wild in the two provinces where our study facilities are located; but nonetheless, the numbers, sizes and fecundities of the animals harvested have not declined. Our data thus strongly support the conclusion from the earlier study, that (despite intuition to the contrary) this enormous offtake is sustainable.

To err on the side of caution, we must explore any bias that might invalidate that conclusion. The only plausible contender is an increase in catching effort and/or the area over which pythons are collected. That is, does constancy in offtake rates between 1995 and 2015 hide a decrease in wild populations that is masked by an increase in collecting effort and area? We see no evidence to support that possibility. First, the value of a python to the collector (the major determinant of collecting effort) has not increased significantly (relative to inflation) between the two periods: roughly $USD 20–30 for a 300 cm animal (approximately 60% of mean weekly income in those regions) [13,28]. Because this system is density-dependent, competition among hunters limits the amount of effort that can be invested searching for snakes. Effort can thus increase only to a certain point before python encounter rates (and thus income) falls to a level where hunters naturally migrate to other employment [22]. As a result, catch effort by “professional” (fulltime) snake-collectors is also likely to have been relatively constant across the last few decades. Minor fluctuations in the numbers of professional collectors are unlikely to have had much impact on overall numbers of snakes collected, because most of the harvested reticulated pythons are encountered by chance as villagers and oil palm workers go about their normal activities, rather than being specifically targeted by professional snake-hunters [7]. Similarly, there is no indication of a shift in the geographic sources of harvested snakes or the habitats in which they are found. Our records of the capture locations of snakes brought to processing facilities show that while some do arrive from far away, most are captured nearby (< 50 km away). Traders claim that snakes are captured from the same areas as 20 years ago, mainly in oil palm estates and secondary forests.

What role does deforestation play in influencing our data? As natural forest is cleared, we might expect displaced snakes to become more vulnerable to capture, which could explain the relative constancy of offtake. However, available data do not support this view. Over the course of our 20-year study period, the deforestation rate in southern and northern Sumatra has declined by 96% and 52%, respectively [29]. If people were capturing large numbers of pythons in the wake of forest clearing, we should thus expect offtake levels to experience a corresponding decrease. The fact that this has not occurred supports the claim that most snakes are captured in oil palm plantations (many of which are > 100 km from the nearest primary forests). The persistence of large snakes in our 2015 sample is similarly intriguing. Presumably, constant harvesting pressure should result in larger (and probably older) snakes becoming less common. If deforestation is important, we would expect greater numbers of large pythons to be brought to facilities in provinces with the highest rates of deforestation. Our conversations with hunters confirm that most large snakes are captured in forested areas (Natusch unpubl. data). Paradoxically, however, large pythons were more common in our sample from Southern Sumatra, where deforestation rates have declined most dramatically (by 94% since our first sampling period [29]). We strongly suspect that detectability of large pythons is related to habitat heterogeneity rather than to habitat type itself (indeed, it is not uncommon to find large reticulated pythons in cities with millions of people–a situation echoed by other large pythonids; Natusch unpubl. data; [30,31]). Thus, accessibility to forested areas (rather than deforestation itself) probably drives this effect, and reflects the larger areas of homogeneous oil palm plantations in northern than southern Sumatra [32]. That being said, unharvested forests (or any habitat type) can act as refuges for python populations, and thus play an important role in enhancing the sustainability of this trade.

But what if our data did indicate that harvesting of reticulated pythons is unsustainable? Would the species be in imminent risk of extinction, or would it merely be the case that the maximum sustainable yield had been exceeded? We suggest the latter interpretation. Reticulated pythons are remarkable generalists in their habitat and dietary needs, and appear to have increased their densities within the oil palm plantations that have expanded substantially within their range [7,32]. The sedentary and cryptic nature of these snakes, which present difficulties to researchers attempting to survey their populations, also impede the ability of hunters to target and collect many animals in one place at one time. Coupled with the result that these giant constrictors are able to sustain intense harvesting, any population decline is likely to be slow. It is thus improbable that trade will result in the extinction of reticulated pythons. If unsustainable trade is detected, the rates of population decline likely would be slow enough to provide authorities with ample time to amend management protocols.

Our findings accord with harvests of other large tropical reptiles. Argentine Tegu lizards (*Tupinambis*) and Yellow Anacondas (*Eunectes notaeus*) also appear to be sustaining large annual harvests. Those species have similar life-history traits to reticulated pythons (rapid maturity, high fecundity; [33,34], which may explain similarities in their ability to persist despite intense hunting pressure. Nevertheless, despite our data suggesting this system is sustainable, it is important to include a cautionary note. Sustainable harvesting of reticulated pythons and other tropical reptiles does not mean that harvests of other snake species will also be sustainable. Tropical snakes grow and reproduce more rapidly than do their temperate relatives [7], and harvesting has caused potentially unsustainable declines in some snake taxa [35,36,37]. Among snakes, pythons may be particularly resilient to harvesting pressure due to high rates of hatching success (because females brood and defend their eggs; [38,39]) and hatchling survival (because of relatively large young; [40]). However, a reliance on sit-and-wait predation may render them vulnerable to habitat degradation that removes potential ambush sites [41].

Repeated surveys of harvested individuals, the focus of our study, likely provide the only robust method for estimating sustainability of the Indonesian offtake of giant reptiles. These ambush-hunting snakes are very difficult to detect in dense tropical forests, because the snakes are inactive for long periods and are exceptionally well camouflaged. Attempting to enumerate underlying reptile abundances in such a system is fraught with difficulty and potential biases [7,42]. The logistical impediments involved in such a task, coupled with environmental variability and difficulties in obtaining sufficiently large samples of snakes, are likely to reduce the power of detecting population changes to unacceptably low levels. In contrast, an analysis of the harvested individuals is straightforward and can provide large sample sizes in short time periods, vastly increasing the statistical power of comparisons and thus confidence that management decisions reflect robust interpretation of results.

Despite the obvious practicality of harvest monitoring, external factors that can influence results must be taken into account if accurate conclusions are to be made about harvest sustainability. For example, a decrease in mean body sizes of harvested snakes may signal a decline in the wild population, but could equally be the result of shifting market trends toward smaller skins (and thus snakes). Our body size data provide a useful example of this, whereby consumer demand for skins above a minimum size has resulted in an increase in the body sizes of snakes harvested today compared to 20 years ago [16].

What can our data tell us to inform future monitoring strategies? Firstly, managers must define what annual level of change they wish to detect and what logistical implications this will have. For example, the minimum sample size needed to accurately detect an annual decline of 10% is 1,238, but increases to 4,947 snakes to detect a change of 5% (Fig 5). Obviously the time required to examine so many snakes is considerable. Thus, we need to consider whether annual monitoring is the optimal survey frequency. If population changes occur slowly (which we expect in this case), it may be more appropriate to sample every 2^nd^ or 3^rd^ year, particularly where resources for such activities are scarce. Because our data from the last 20 years suggest sustainability has been achieved, managers should aim to conduct monitoring surveys every 2^nd^ or 3^rd^ year (but more regularly if possible). The minimum sample sizes required to detect change with sufficient statistical power would be 551 individual snakes at each site (to detect a cumulative change of 15% over three years, or 5% annually; Fig 5). Such a sampling protocol would serve the dual benefit of minimising the costs of monitoring while improving confidence that any observed change is harvest-related rather than due to environmental effects or survey biases.

Perhaps surprisingly, our results may also have implications for invasive species management. The introduction of large constrictors into the southern United States has been implicated in the decline of several native species, prompting intense research into ways to control and mitigate these impacts [43,44]. A novel approach to this problem has been the establishment of the “Python Challenge”, a 60-day event whereby hunters are encouraged to kill Burmese Pythons (*Python molurus*) in an attempt to control snake numbers and raise awareness about invasive species [45]. Our result that, despite almost a century of intensive harvesting, reticulated pythons are still harvested in enormous numbers suggests this kind of limited culling will likely have no effect on invasive populations of Burmese Pythons.

Our results are largely positive about the sustainability of reticulated python harvests in Indonesia. Nonetheless, sustainability can only reliably be determined in hindsight and we stress the need for ongoing monitoring of the Indonesian (and Malaysian) wild harvests of reticulated pythons. Our study contradicts assumptions of unsustainable trade (which have resulted in trade restrictions), and underpins the need for robust science to inform wildlife trade policy and decision-making. Although inferences based on attributes of the commercial harvest inevitably are indirect, the logistical advantages of the approach mean that an ongoing program of monitoring–preferably combined with information on harvest locations and catching effort–will allow for adaptation of management regimes should harvest-related changes to the population be detected. Such a straightforward yet robust approach to sustainability assessments is applicable to harvests of many other species of snakes, and indeed many other harvested taxa inhabiting tropical countries around the world.
